# Moderate Exercise Suppresses NF-κB Signaling and Activates the SIRT1-AMPK-PGC1α Axis to Attenuate Muscle Loss in Diabetic *db/db* Mice

**DOI:** 10.3389/fphys.2018.00636

**Published:** 2018-05-29

**Authors:** Hung-Wen Liu, Sue-Joan Chang

**Affiliations:** ^1^Department of Physical Education, National Taiwan Normal University, Taipei, Taiwan; ^2^Department of Life Sciences, National Cheng Kung University, Tainan, Taiwan

**Keywords:** MuRF-1, moderate exercise, NF-κB signaling, mitochondrial function/biogenesis, diabetic *db/db* mice

## Abstract

The clear mechanism of moderate exercise training (Ex) in attenuating muscle loss remains elusive in diabetes. We investigated the effects of moderate exercise training on diabetes-induced nuclear factor-κB (NF-κB) activation and mitochondrial dysfunction. Skeletal muscle size and atrophy signaling pathways were examined in type 2 diabetic *db/db* mice with or without moderate exercise training (5.2 m/min, 1 h/day, and 5 days/week for a total of 8 weeks). Exercise training decreased serum leptin, MCP-1, and resistin levels in *db/db*+Ex mice, but it did not reduce symptoms of insulin resistance including hyperglycemia, hyperinsulinemia, and impaired glucose tolerance. Moderate exercise training prevented the loss of muscle mass of tibialis anterior and gastrocnemius muscles in *db/db*+Ex mice. The average cross-sectional area of tibialis anterior muscle was increased significantly in *db/db*+Ex mice compared with untrained mice (830.6 vs. 676.5 μm^2^). Inhibition of MuRF-1 and K48-linked polyubiquitination was observed in *db/db*+Ex mice. Exercise training reduced activation of IκBα/NF-κB pathway and lowered IL-6, TNFα, F4/80 (macrophage marker) at mRNA level in *db/db*+Ex mice compared with untrained mice. Exercise training did not influence FoxO3a phosphorylation and its upstream regulator Akt. Exercise training increased SIRT1 and PGC1α expression and AMPKα and mitochondrial complex IV activities and upregulated genes involved in mitochondrial biogenesis/function including Nrf1, Tfam, and mitochondrial complexes I–V. In conclusion, moderate exercise training inhibits NFκB signaling and activates SIRT1-AMPKα-PGC1α axis, thereby attenuating type 2 diabetes-related muscle atrophy.

## Introduction

Certain metabolic disorders such as diabetes and obesity are involved in the development of muscle atrophy, a decrease in the mass of skeletal muscle (Kalyani et al., 2014; Le et al., 2014). It is recognized that activation of ubiquitin-proteasome proteolytic pathways induced by chronic inflammation is involved in the pathogenesis of muscle atrophy (Cai et al., 2004; Le et al., 2014; Costamagna et al., 2015). MuRF-1, a muscle specific E3 ubiquitin ligases is an important regulator of ubiquitin-mediated protein degradation in skeletal muscle (Bodine et al., 2001). Transcriptional factors, NF-κB and FoxO3a translocate into nucleus and subsequently upregulate transcriptional activities of MuRF-1 under certain pathological conditions (Cai et al., 2004; Gumucio and Mendias, 2013; Perry et al., 2016). This suggests that inhibition of NF-κB and/or FoxO3a pathways is a promising target for preventing muscle atrophy. In addition, mitochondrial dysfunction accompanied by generation of excessive reactive oxygen species contributes to elevated oxidative stress (Zorov et al., 2014) that leads to impaired skeletal muscle function and accelerated loss of muscle mass in many diseases such as disuse/inactivity, diabetes, cancer, and sarcopenia (Romanello and Sandri, 2015; Joseph et al., 2016). Therefore, restoration of mitochondrial function is critical for maintaining skeletal muscle homeostasis.

Beneficial effects of aerobic exercise training on age-related muscle loss have been widely recognized (Short et al., 2004; Kalyani et al., 2014). Only few studies so far have investigated the molecular basis of the beneficial effects of aerobic exercise training on the skeletal muscle health, particularly muscle proteolysis, in diabetes models. Aerobic exercise training-induced downregulation of MuRF-1 expression has been addressed (Chen et al., 2011; Ostler et al., 2014); however, the clear mechanism by which exercise training modulates atrophy signaling pathways in diabetes remains unclear. In *db/db* mice, high-intensity exercise (15 m/min for 30 min for 12 weeks) was related to elevated plasma cortisol levels (Sennott et al., 2008), a catabolic hormone involved in activation of protein degradation pathway. For this reason, mild-intensity exercise is a more appropriate for type 2 diabetes-related muscle atrophy.

In the present study, *db/db* mouse, a popular murine model of obesity and type 2 diabetes, was used to examine effects of exercise training on regulation of MuRF-1 and its transcriptional regulators NF-κB and FoxO3a. Additionally, we investigated whether exercise training-mediated upregulation of SIRT1-AMPKα-PGC1α (SIRT1-AMPKα-PGC1α) axis and mitochondrial biogenesis could facilitate regulation of muscle mass. We hypothesized that moderate exercise training decreases NF-κB activation and promotes mitochondrial adaptations, thus preventing skeletal muscle protein degradation in type 2 diabetes.

## Materials and Methods

### Materials

Primary antibodies: Akt (#9272), phospho-Akt (Thr308) (#4056), FoxO3a (#2497), phospho-FoxO3a (Ser253) (#9466), AMPKα (#2603), phospho-AMPKα (Thr172) (#2535), IκBα (#4814), phosphor-IκBα (Ser32) (#2859), K48-linkage polyubiquitin (#4289), NF-κB p65 (#4764), phospho-NF-κB p65 (Ser563) (#3033), and SIRT1 (#3931), were purchased from Cell Signaling (Danvers, MA, United States). PGC1α (ab54481) was purchased from Abcam (Cambridge, MA, United States). MuRF-1 (sc-398608) and GAPDH (sc-47724) were purchased from Santa Cruz Biotechnology (Dallas, TX, United States). Goat anti-rabbit (#7074) and horse anti-mouse (#7076) HRP conjugated secondary antibodies were purchased from Cell signaling (Danvers, MA, United States).

### Experimental Animals

Animal experiments were approved by the National Taiwan Normal University Institutional Animal Care and Use Committee (Approval Number: 105017). Four-week-old male diabetic C57BLKS/J (*db/db*) mice (*n* = 24) and their age-matched corresponding control (*m/m*, *n* = 12) mice were purchased from the National Laboratory Animal Center (Taipei, Taiwan). Two or three mice per cage were housed in an air-conditioned animal facility at 20 ± 2°C, 50 ± 5% humidity, and 12 h light/dark cycle with free access to water and normal chow diet (LabDiet 5058, St. Louis, MO, United States). Body weight was measured weekly. At the age of 5 weeks, *db/db* mice were divided into two groups: *db/db* mice (*n* = 12) with moderate exercise training for 8 weeks and *db/db* (*n* = 12) remained sedentary throughout the study. Animals were anesthetized by intraperitoneal injection of urethane (1500 mg/1 kg BW) followed by decapitation between 10 to 12 am. Trunk blood was collected from overnight fasted mice in non-heparinized tubes. Serum was separated by centrifugation at 3,000 rpm for 15 min and stored at -20°C. Wet muscles were removed, briefly rinsed with PBS, removed excess fluid, and weighed on a digital balance. Tibialis anterior muscles were fixed with 4% paraformaldehyde and gastrocnemius muscles were stored at -80°C for further analysis.

### Moderate Exercise Training

Moderate exercise training used in the present study has been shown to attenuate diabetes-induced renal disease, coronary vascular dysfunction, and vascular endothelial dysfunction in *db/db* mice (Moien-Afshari et al., 2008a,b; Ghosh et al., 2009). Eight weeks moderate-intensity exercise (5.2 m/min, 1 h/day, and 5 days/week for a total of 8 weeks) was started from 5-week-old. During the 1st week, mice ran on a motorized treadmill (30 min with 0° slope) and exercise duration was gradually increased from 30 min to the target of 1h (0° slope). *db/db* and *m/m* mice remained sedentary were placed on the treadmill belt for the same duration.

### Intraperitoneal Glucose Tolerance Test (IPGTT) and Serum Biological Markers Measurement

IPGTT test (*n* = 6/group) was performed 2 days after the last training day. Overnight fasted (12 h) mice were given glucose (1 g/kg BW) via intraperitoneal injection. Blood samples were taken by tail snipping at 0, 15, 30, 60, and 120 min after glucose injection. Blood glucose levels were measured by ACCU-CHEK (Roche, Basel, Switzerland). Two days later, overnight fasted mice were euthanized for tissue and serum collection. Serum insulin, leptin, MCP-1, resistin, IL-6, and TNFα were measured by Milliplex map kit (Millipore, Billerica, MA, United States).

### Skeletal Muscle Histology

Embedded tibialis anterior muscle blocks from *m/m*, *db/db*, and *db/db*+Ex groups (*n* = 4/group) were cut into 5 μm sections and stained with hematoxylin-eosin. Images were observed under a microscope and captured with a digital camera (Olympus, Tokyo, Japan). Average muscle fiber CSA of tibialis anterior muscle (3 randomly selected images per animal) was determined using ImageJ with careful manual annotations and 20–45 fibers per image were counted.

### Western Blot Analyses

Gastrocnemius muscle was cut into small pieces and homogenized in ice-cold RIPA buffer containing 1 mM phenylmethylsulfonyl fluoride and protease inhibitor cocktail (Millipore, Billerica, MA, United States). Total protein in the homogenate was measured by the Bradford dye-binding method (Bio-Rad, Hercules, CA, United States). Homogenates of gastrocnemius muscles were separated by SDS-PAGE, transferred to nitrocellulose membrane, and incubated with appropriate antibodies. Protein bands were visualized using Chemiluminescence kit (Millipore, Billerica, MA, United States) and quantified by using the LAS-4000 mini biomolecular imager (GE HealthCare Life Sciences, Pittsburgh, PA, United States).

### RNA Extraction and Real-Time PCR

Total RNA was extracted from gastrocnemius muscle by using Trizol/chloroform procedure (Thermo Fisher Scientific, Waltham, MA, United States) and quantified by the NanoDrop meter. Total RNA (1 μg) was reverse transcribed into cDNAs by using cDNA synthesis kit (Bio-Rad, Hercules, CA, United States). Real-time PCR was performed using SYBR Green Master Mix kit (Applied Biosystems, Foster City, CA, United States). The PCR reaction included the following components: each primer at a concentration of 10 μM, cDNA template (16 ng), and SYBR Green Master Mix and running 40 cycles. Each cDNA sample was run in triplicate and 18s primers as an internal control were included in each run to correct sample to sample variation and to normalize mRNA levels. The relative mRNA level was quantified using StepOnePlus Real-Time system (Applied Biosystems, Foster City, CA, United States). Fold change expression was calculated according to the comparative ΔΔ*C*T method. Primer sequences are shown in **Table 1**.

**Table 1:** Real-time PCR primers.

Genes	GenBank accession	Forward (5′→3′)	Reverse (5′→3′)
Atp5a1	NM_007505	TCTCCATGCCTCTAACACTCG	CCAGGTCAACAGACGTGTCAG
Cox5b	NM_009942	TTCAAGGTTACTTCGCGGAGT	CGGGACTAGATTAGGGTCTTCC
F4/80	NM_001355722	TTGTACGTGCAACTCAGGACT	GATCCCAGAGTGTTGATGCAA
IL6	NM_031168	CTCTGGGAAATCGTGGAAAT	CCAGTTTGGTAGCATCCATC
Ndufs8	NM_144870	AGTGGCGGCAACGTACAAG	TCGAAAGAGGTAACTTAGGGTCA
Nrf1	NM_001164230	AGCACGGAGTGACCCAAAC	TGTACGTGGCTACATGGACCT
Sdhb	NM_023374	AATTTGCCATTTACCGATGGGA	AGCATCCAACACCATAGGTCC
Tfam	NM_009360	ATTCCGAAGTGTTTTTCCAGCA	TCTGAAAGTTTTGCATCTGGGT
TNFA	NM_013693	ATGAGAAGTTCCCAAATGGC	CTCCACTTGGTGGTTTGCTA
Uqcrc1	NM_025407	AGACCCAGGTCAGCATCTTG	GCCGATTCTTTGTTCCCTTGA
18S	NR_003278	GGGAGCCTGAGAAACGGC	GGGTCGGGAGTGGGTAATTT

### Cytochrome Oxidase (Complex IV) Activity

Crude mitochondrial fraction was extracted from gastrocnemius muscle (25 mg) using mammalian mitochondrial isolation kit (BioVision, Milpitas, CA, United States, catalog#K288) followed by manufacturer’s instructions. Protein concentration was measured by the Bradford dye-binding method (Bio-Rad, Hercules, CA, United States). Mitochondrial extract (5 μg) was mixed with reduced cytochrome c and then immediately read at 550 nm for 30 min at 30 s interval. Cytochrome oxidase activity was calculated following manufacturer’s instructions (BioVision, Milpitas, CA, United States, catalog#K287).

### Statistical Analysis

Data are expressed as means ± SEM. The statistical significance of the differences among *m/m*, *db/db*, and *db/db*+Ex groups was determined by one-way ANOVA and following *post hoc* assessment by Student-Newman–Keuls Method correction for multiple comparisons (SigmaPlot 12.0, San Jose, CA, United States). Different lowercase letters indicate significant differences among groups. The student’s *t*-test was used to determine the statistical significance of the differences between *db/db* and *db/db*+Ex groups (SigmaPlot 12.0). A *P*-value less than 0.05 was considered to be statistically significant.

## Results

### Effects of Exercise on Body Weight, Blood Glucose, Insulin, Glucose Tolerance, and Serum Biological Markers

**FIGURE 1:**
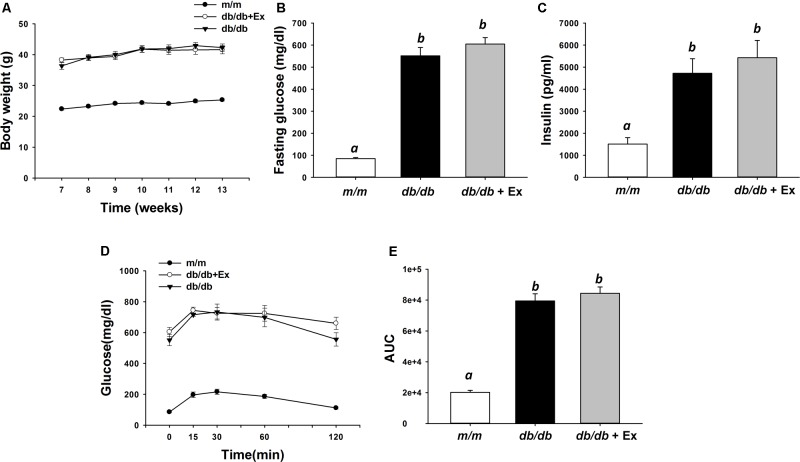
Effect of moderate exercise training on body weight body, blood glucose, insulin, and glucose tolerance. Body weight (**A**, *n* = 11–12/group), fasting glucose (**B**, *n* = 6/group), serum insulin (**C**, *n* = 11–12/group), IPGTT (**D**, *n* = 6/group), and area under the curve (AUC) calculations (**E**, *n* = 6/group) in *m/m*, *db/db*, and *db/db*+Ex groups. Values presented are mean ± SEM. Significance (*P* < 0.05) among groups is denoted by different letters.

Body weight in *db/db* and *db/db*+Ex groups were higher than *m/m* mice throughout the experiment (**Figure 1A**). Significantly increased fasting glucose and serum insulin levels were observed in *db/db* and *db/db*+Ex compared with *m/m* mice (**Figures 1B,C**). Glucose tolerance was examined by area under the curve (AUC) during IPGTT (**Figure 1D**). Impaired glucose tolerance was observed in *db/db* and *db/db*+Ex groups compared with *m/m* mice (**Figures 1D,E**). Significantly decreased leptin, MCP-1, and resistin levels were observed in *db/db* mice after moderate exercise training compared with the non-exercised group (**Table 2**). Leptin, MCP-1, and resistin levels in *db/db*+Ex mice were 8.6, 75, and 39% lower, respectively, compared with *db/db* mice without exercise training.

**Table 2:** Serum adipokines and chemokine.

pg/ml	*m/m*	*db/db*	*db/db*+Ex
Leptin	718.8 ± 100.5^a^	6640.7 ± 306.3^b^	5705.4 ± 222.0^c^
MCP-1	1.8 ± 0.4^a^	85.6 ± 41^b^	21.3 ± 9.4^a^
Resistin	689.0 ± 68.1^a^	978.2 ± 120^b^	593.6 ± 69.3^a^

*Values are mean ± SEM. Significance (P < 0.05) among groups is denoted by different letters*.

### Effects of Exercise on Muscle Fiber Size, Muscle Weight, MuRF-1 and Ubiquitination

**FIGURE 2:**
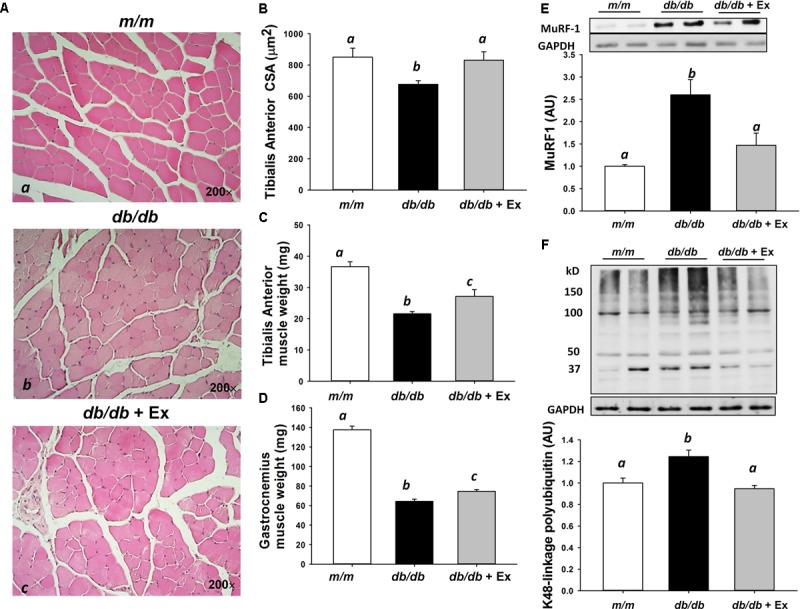
Effect of moderate exercise training on muscle size, muscle weight, and protein expression. Representative hematoxylin-eosin stained of tibialis anterior muscle **(Aa–c)** in *m/m*, *db/db*, and d*b/db*+Ex groups (200×). Mean cross-sectional area of tibialis anterior muscle (**B**, *n* = 4/group). Tibialis anterior **(C)** and gastrocnemius **(D)** muscle weight (*n* = 9–12/group). Representative blots of MuRF-1 and K48-linkage polyubiquitin (**E,F**, *n* = 5–8). Values presented are mean ± SEM. Significance (*P* < 0.05) among groups is denoted by different letters.

The average CSA of tibialis anterior muscle was reduced significantly in *db/db* mice compared with *m/m* mice (**Figures 2A,B**). The average CSA was increased significantly in *db/db* mice after exercise training (**Figures 2A,B**). Muscle weights of tibialis anterior and gastrocnemius muscles in *m/m* mice were greater than *db/db* mice (**Figures 2C,D**). In contrast to *db/db* mice without exercise training, muscle weights of tibialis anterior and gastrocnemius muscles were increased by 26 and 16%, respectively, after moderate exercise training (**Figures 2C,D**). Protein levels of muscle-specific E3 ubiquitin-ligase, MuRF-1, and ubiquitin (K48-linakage polyubiquitin)-conjugated proteins were used to determine the ubiquitin-proteasome system activity. Increased MuRF-1 and K48-linakage polyubiquitin levels were markedly suppressed in *db/db* mice after moderate exercise training (**Figures 2E,F**).

### Effects of Exercise on NF-κB and FoxO3a Signaling Pathways

**FIGURE 3:**
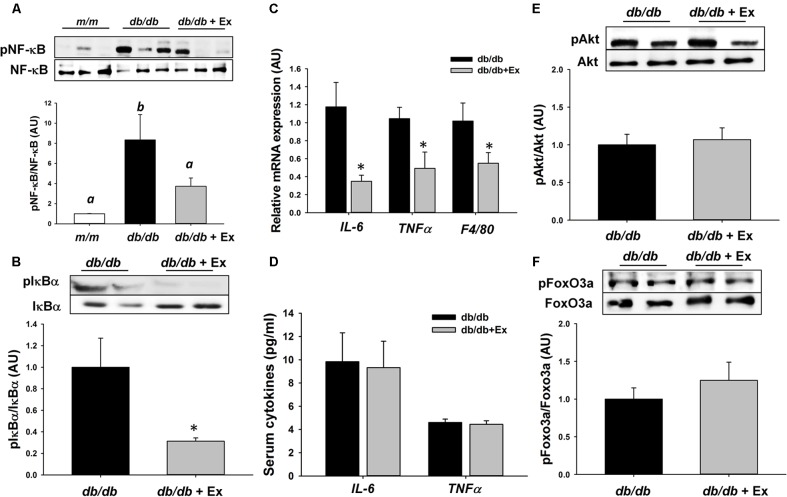
Effect of moderate exercise training on protein expression, mRNA expression, and proinflammatory cytokines. Representative blots of NF-κB, phospho-NF-κB (Ser563), IκBα, and phospho-IκBα (Ser32), are shown **(A,B)**. The protein levels in gastrocnemius muscles are presented mean ± SEM (*n* = 5/group). IL-6, TNFα, and F4/80 mRNA expression levels in gastrocnemius muscles are expressed as mean ratio to control after normalization with 18S mRNA levels. Fold differences were calculated using the ΔΔ*C*t method. Values presented are mean ± SEM (**C**, *n* = 6–8/group, each in triplicate). Serum levels of IL-6 and TNFα (**D**, *n* = 11–12/each group). Representative blots of Akt, phospho-Akt (Thr308), FoxO3a, and phospho-FoxO3a (Ser253) are shown **(E,F)**. The protein levels in gastrocnemius muscles are presented mean ± SEM (*n* = 5–7/group). Significance (*P* < 0.05) among groups is denoted by different letters. ^∗^*P* < 0.05 vs. *db/db*.

Activation of IκBα via phosphorylation results in the release and nuclear translocation of active NF-κB, leading to upregulation of atrophy-related and proinflammatory genes under atrophic conditions. Phosphorylation of NF-κB and IκBα was reduced significantly in *db/db*+Ex mice compared with *db/db* mice (**Figures 3A,B**). Inflammatory genes including IL-6 and TNFα were markedly suppressed by moderate exercise training (**Figure 3C**). Furthermore, suppression of macrophage marker F4/80 at mRNA level was observed in *db/db*+Ex mice (**Figure 3C**). Exercise training did not affect serum IL-6 and TNFα levels in both groups (**Figure 3D**). FoxO3a is also involved in the transcriptional regulation of MuRF-1 expression. There was no difference in phosphorylation FoxO3a and its upstream regulator Akt between *db/db* and *db/db*+Ex groups (**Figures 3E,F**).

### Effects of Exercise on SIRT1-AMPKα-PGC1α Axis and Mitochondrial Adaptations

**FIGURE 4:**
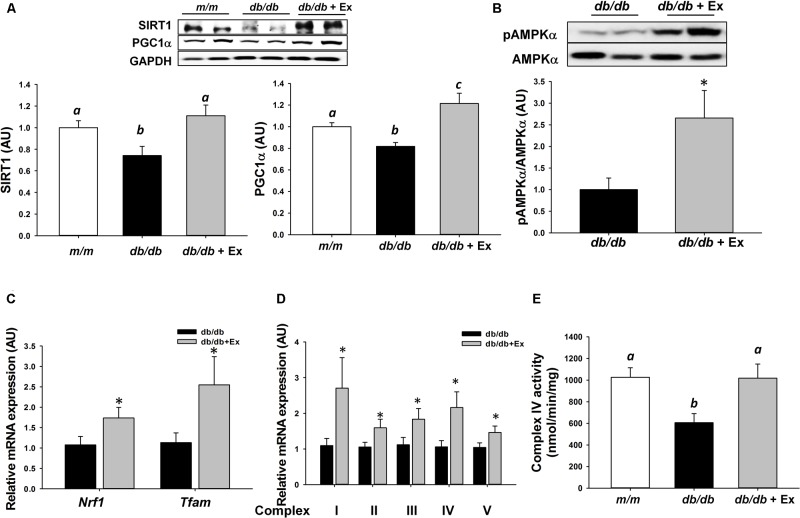
Effect of moderate exercise training on protein expression, mRNA expression, and complex IV activity. Representative blots of SIRT1, PGC1α, AMPKα, and phospho-AMPKα (Thr172) are shown **(A,B)**. The protein levels in gastrocnemius muscles are presented mean ± SEM (*n* = 5–7/group). Nrf1, Tfam, and mitochondrial complexes I–V mRNA expression levels in gastrocnemius muscles are expressed as mean ratio to control after normalization with 18S mRNA levels. Fold differences were calculated using the ΔΔ*C*t method. Values presented are mean ± SEM (**C,D**, *n* = 6–8/group, each in triplicate). Cytochrome oxidase (Complex IV) activity in gastrocnemius muscle (**E**, *n* = 6/group). Significance (*P* < 0.05) among groups is denoted by different letters. ^∗^*P* < 0.05 vs. *db/db*.

Mitochondrial dysfunction stimulates catabolic signaling pathways and subsequently promotes skeletal muscle atrophy. SIRT1, AMPKα, and PGC1α, key regulators of energy metabolism are required for exercise training-induced increases in mitochondrial function and biogenesis. Moderate exercise training significantly increased SIRT1 and PGC1α expression and AMPKα phosphorylation in *db/db*+Ex mice (**Figures 4A,B**). Nrf1, Tfam, subunits of mitochondrial complexes I–V at mRNA level, and mitochondrial complex IV activity were increased significantly in response to moderate exercise training (**Figures 4C–E**).

## Discussion

Increased muscle protein degradation is associated with disruption of the homeostatic regulation of muscle mass in diabetes (Chen et al., 2011; Workeneh and Bajaj, 2013; Ostler et al., 2014). Catabolic factors such as NF-κB activation and abnormal production of inflammatory cytokines stimulate ubiquitin-proteasome proteolytic pathway (Costamagna et al., 2015). Regular aerobic exercise has been long known in the prevention and management of type 2 diabetes (Colberg et al., 2010). The clear mechanisms of aerobic exercise training on the regulation of muscle protein degradation in diabetes model remain elusive. Here, exercise effects on NF-κB and FoxO3a signaling pathways which are involved in regulation of MuRF-1 expression have been examined in diabetic *db/db* mice. Furthermore, exercise effects on SIRT1, AMPKα, PGC1α, and mitochondria-related genes were also investigated in skeletal muscle. We demonstrated that moderate exercise training successfully inhibited muscle atrophy-related ubiquitin ligase, MuRF-1, in association with suppression of NF-κB and activation of SIRT1, thereby attenuating protein degradation in diabetes.

Beneficial effects of treadmill exercise training on decreased MuRF-1 expression as well as increased muscle weight in diabetic rodent models have been demonstrated by several studies (Chen et al., 2011; Ostler et al., 2014). Our data were consistent with previous studies showing that exercise inhibited ubiquitin-mediated protein degradation in skeletal muscle via suppression of MuRF-1. To further understand the anti-atrophy effect of exercise training, NF-κB signaling and pro-inflammatory cytokines, IL-6 and TNFα, were investigated. Here, moderate exercise training suppressed NF-κB signaling and its pro-inflammatory target genes, IL-6 and TNFα in skeletal muscle of diabetic *db/db* mice. Nevertheless, moderate exercise training was not able to lower serum IL-6 and TNFα levels. Suppressed adaptive immunity including inhibition of cytokine secretion was observed in *db/db* mice, which could explain no changes in serum IL-6 and TNFα levels between *db/db* and *db/db*+Ex groups. Exercise stimulates NF-κB activation (Kramer and Goodyear, 2007; Tantiwong et al., 2010), especially during high-intensity exercise (Cuevas et al., 2005). For this reason, *db/db* mice performed moderate-intensity exercise training (5.2 m/min, 1 h/day) to prevent over-activation of NF-κB signaling. In this context, inflammatory responses-induced protein degradation would not be exacerbated by exercise training. In the present study, moderate exercise training is sufficient to effectively suppress NF-κB signaling and inflammation-related genes. Also, *db/db*+Ex mice displayed significantly less macrophage infiltration, demonstrated by lower F4/80 expression and MCP-1 levels. NF-κB activation plays an important role in fast-twitch fiber atrophy under atrophic conditions (Wang and Pessin, 2013). Attenuation of muscle loss in fast-type skeletal muscle (gastrocnemius and tibialis anterior) by exercise was related to suppression of NF-kB signaling. Hence, moderate exercise training is recommended for patients with type 2 diabetes to attenuate muscle loss.

FoxOs members are important transcriptional factors involved in the regulation of MuRF-1 expression (Leger et al., 2006; Milan et al., 2015). In the present study, phosphorylation of FoxO3a and its upstream Akt were not affected by moderate exercise training. *db/db*+Ex mice still exhibited hyperglycemia, hyperinsulinemia, and impaired glucose tolerance, indicating that moderate exercise training was not able to improve insulin sensitivity. These results are consistent with previous studies employing the same exercise training program (Moien-Afshari et al., 2008a,b; Ghosh et al., 2009). Taken together, our data suggest that the beneficial effect of moderate exercise training on skeletal muscle health is mediated through inhibition of NF-κB-mediated inflammation rather than through direct management of whole-body insulin sensitivity.

SIRT1 inhibits NF-κB activity directly through deacetylation (Yeung et al., 2004) and/or coordinates multiple signaling pathways including AMPK, PGC-1α (peroxisome proliferator-activated receptor gamma coactivator-1α, and PPARα peroxisome proliferator-activated receptor α) to suppress NF-κB signaling (Kauppinen et al., 2013). In the present study, suppression of NFκB signaling was likely mediated, in part, through exercise-mediated upregulation of SIRT1-AMPK-PGC1α axis. Although direct evidence is not based on exercise intervention, it has been demonstrated that anti-inflammatory compounds such as isoflavones and flavonols prevent MuRF-1-mediated muscle atrophy via suppression of NF-κB and activation of SIRT1 in C2C12 myotubes (Hirasaka et al., 2013) and skeletal muscle of *db/db* mice (Liu et al., 2017). Therefore, SIRT1-AMPK-PGC1α axis may be a therapeutic target in inflammation-induced loss of skeletal muscle.

Mitochondrial dysfunction has been implicated in the development of disuse-induced muscle atrophy and age-related muscle loss (Powers et al., 2012; Joseph et al., 2016). Mitochondrial dysfunction is frequently observed in skeletal muscle in type 2 diabetes (Kelley et al., 2002; Montgomery and Turner, 2015); suggesting that restoration of mitochondrial function may help type 2 diabetic patients improve their muscle health. We showed that moderate exercise training increased SIRT1 and PGC1α expression, stimulated AMPKα activation, and subsequently upregulated Nrf1, Tfam, subunits of mitochondrial complexes at mRNA level, and mitochondrial complex IV activity in diabetic *db/db* mice. Results from the present study support the hypothesis that exercise-induced mitochondrial adaptations contribute to maintaining muscle homeostasis.

The main purpose of the study is to explore the underlying mechanism of moderate exercise training on the regulation of muscle protein degradation in type 2 diabetes; nevertheless, the diabetic model of *db/db* has its own advantages and limitations. A previous study demonstrated that insulin resistance and chronic hyperglycemia alone were not sufficient to rapidly increase muscle proteolysis by comparing two models of type 2 diabetes (*db/db* mice vs. TallyHo mice) (Ostler et al., 2014). A markedly elevated corticosterone level in *db/db* mice could be the key mediator involved in activation of protein degradation pathway (Ostler et al., 2014). In the present study, suppressed activation of protein degradation pathway in exercising *db/db* group may be associated with low levels of glucocorticoids. Although exercise prevents skeletal muscle protein degradation, exercise alone is insufficient to fully rescue muscle mass in leptin receptor deficient diabetic (*db/db*) mice. Impaired myoblast proliferation and differentiation in mice lacking of functional leptin receptor isoforms indicates the importance of leptin receptor in the regulation of muscle mass (Arounleut et al., 2013). The diabetic *db/db* mice have less muscle mass compared to their age-matched corresponding control due to impairment of muscle growth and development, and that a limitation of the *db/db* mouse in translational the muscle atrophy research.

Loss of muscle mass can be triggered by certain pathological conditions including type 2 diabetes. Reduced muscle mass in patients with type 2 diabetes impairs their muscle function and may increase the risk of mortality (Perry et al., 2016). Inhibition of NF-κB, MuRF-1, and ubiquitin-mediated protein degradation by moderate exercise was observed in *db/db* mice. These findings provides better understand the anti-inflammatory effect of exercise training in type 2 diabetes-related muscle atrophy. Evidence from animal research supports the clinical use of moderate exercise to improve muscle health.

## Conclusion

In summary, moderate exercise training decreases NF-κB activation and represses inflammatory gene expression. Furthermore, exercise training triggers activation of SIRT1-AMPK-PGC1α axis and increases markers of mitochondrial biogenesis and mitochondrial complex IV activity. Exercise-mediated decrease in catabolic factors is associated with preventing activation of the ubiquitin-proteasome proteolysis pathway. In type 2 diabetes, lifestyle modification such as moderate exercise may help maintain muscle health.

## Author Contributions

H-WL and S-JC contributed to the conception and design of the research. H-WL carried out the animal experiments and collected data. The major contribution for writing of this manuscript was from H-WL.

## Conflict of Interest Statement

The authors declare that the research was conducted in the absence of any commercial or financial relationships that could be construed as a potential conflict of interest.

## Abbreviation

AMPKα: AMP-activated protein kinase α
CSA: cross-sectional area
FoxO3a: forkhead box O3
IL-6: interleukin 6
IPGTT: intraperitoneal glucose tolerance test
MCP-1: monocyte chemoattractant protein-1
mTOR: mammalian target of rapamycin
MuRF-1: muscle RING-finger protein-1
NF-κB: nuclear factor-κB
Nrf1: nuclear respiratory factor 1
PGC1α: peroxisome proliferator-activated receptor gamma coactivator 1-α
PPARα: peroxisome proliferator-activated receptor α
SIRT1: sirtuin 1
Tfam: mitochondrial transcription factor A
TNFα: tumor necrosis factor α.
